# Current and Future Potential of Shellfish and Algae Mariculture Carbon Sinks in China

**DOI:** 10.3390/ijerph19148873

**Published:** 2022-07-21

**Authors:** Qiuying Lai, Jie Ma, Fei He, Aiguo Zhang, Dongyan Pei, Minghui Yu

**Affiliations:** 1 Nanjing Institute of Environmental Sciences, Ministry of Ecology and Environment, Nanjing 210042, China; laiqiuying@nies.org (Q.L.); majie@nies.org (J.M.); zhangaiguo@nies.org (A.Z.); 2 School of Environmental Science and Engineering, Nanjing University of Information Science and Technology, Nanjing 210044, China; 20201248123@nuist.edu.cn; 3 College of Environment, Hohai University, Nanjing 210024, China; 211605010035@hhu.edu.cn

**Keywords:** carbon sink, blue carbon, shellfish, algae, carbon neutrality, China

## Abstract

Shellfish and algae mariculture make up an important part of the marine fishery carbon sink. Carbon sink research is necessary to ensure China achieves its goal of carbon neutrality. This study used the material quality assessment method to estimate the carbon sink capacity of shellfish and algae. Product value, carbon storage value, and oxygen release value were used to calculate the economic value of shellfish and algae carbon sequestration. The results showed that the annual average shellfish and algae carbon sink in China was 1.10 million tons from 2003 to 2019, of which shellfish accounted for 91.63%, wherein *Crassostrea* *gigas*, *Ruditapes* *philippinarum*, and *Chlamys* *farreri* were the main contributors. The annual average economic value of China’s shellfish and algae carbon sequestration was USD 71,303.56 million, and the product value was the main contributor, accounting for 99.11%. The carbon sink conversion ratios of shellfish and algae were 8.37% and 5.20%, respectively, thus making shellfish the aquaculture species with the strongest carbon sink capacity and the greatest carbon sink potential. The estimated growth rate in the shellfish and algae removable carbon sink was 33,900 tons/year in China, but this trend was uncertain. The capacity for carbon sequestration and exchange by aquaculture can be improved by expanding breeding space, promoting multi-level comprehensive breeding modes, and marine artificial upwelling projects.

## 1. Introduction

Global warming is one of the most pressing challenges faced by the sustainable development of human societies, and its root cause is carbon emissions [1]. The Paris Agreement sets a long-term temperature goal of holding the global average temperature increase to well below 2 °C, and efforts are underway to limit this to 1.5 °C above pre-industrial levels [2,3]. Global strategies to achieve the goal of carbon neutralization involve both reducing emissions and increasing CO_2_ sinks [4].

The ocean is the largest carbon sink on Earth and plays an important role in regulating global climate change [5]. Marine carbon sequestration involves various processes, activities, and mechanisms that absorb CO_2_ from the atmosphere and fix it in the ocean, retained in marine sediments and organisms. The mechanisms driving the marine carbon sink mainly include physical and biological pumps. The physical pump is mainly dependent on natural processes, e.g., ocean currents, wind, pH, etc., and there is little potential to increase carbon flux through artificial means [6,7]. The biological pump refers to the biological activities that affect “blue carbon”, the carbon stored in marine ecosystems, a system where there is great potential for artificial development. To date, biological carbon storage mechanisms have been broken down into two major categories: the biological pump (BP) and microbial carbon pump (MCP) [8,9,10]. Based on the biological carbon storage mechanisms underlying BP and MCP, good progress has been made in technologies designed to enhance carbon sinks, such as increasing the fishery carbon sink [11,12].

Fisheries have integrated biological carbon sequestration strategies that improve their carbon sink function and emphasize the development of a low-carbon economy [13,14]. Marine primary production is a process in which photosynthetic marine organisms assimilate CO_2_ into organic matter using light energy. As a primary producer, algae are the initial link and a key component of the marine carbon cycle [15]. Algae convert inorganic carbon from seawater into organic carbon through photosynthesis and absorb nutrients to build their own structures [16]. Algae play important roles in the oceanic carbon sink in two ways. Firstly, through the absorption of dissolved CO_2_, algae can reduce the partial pressure of CO_2_, shifting the carbon chemical equilibrium and accelerating the dissolution of atmospheric CO_2_ into seawater. Secondly, the absorption of nutrients by algae during growth can improve the pH of surface seawater in an aquaculture area and reduce the partial pressure of CO_2_, further facilitating the diffusion of atmospheric CO_2_ into seawater. Similarly, filter-feeding shellfish acquire carbon from the ocean through calcification and feeding [17,18]. The marine fishery is essentially an “industrialized blue carbon” pool that serves as a “removable carbon sink”; that is, it promotes the biological absorption of CO_2_ in water bodies using fishery activities and effectively removes that carbon upon the harvesting of the mariculture organisms. Today, numerous assessment methods exist to quantify removable carbon sinks, including the material quality assessment method [19], remote sensing estimation method [20], model simulation method [21], etc., among which the material quality assessment method is widely used because of its convenience of application and high accuracy. In addition to their carbon sink functions, the culture of marine shellfish and algae also serve environmental supply and regulation functions. Most studies accounting for the economic value accounting of carbon sequestration have focused on the narrow concept of a carbon sink’s economic value, that is, the market value of CO_2_ storage increment generated by marine biological, abiotic, and other marine activities. Generally, the product value and oxygen release value have been ignored [22,23]. As an ecological resource, the value composition of a marine fishery carbon sink has much in common with the production value composition of an ecosystem [24,25], and its product value, carbon storage value, and oxygen release value should be considered an inseparable whole.

China’s mariculture industry is the largest in the world and plays an important role in coping with climate change. In the present study, first, the carbon sink capacities of shellfish and algae were estimated using the material quality assessment method based on fishery statistics in China from 2003 to 2019. Second, an economic value accounting of shellfish and algae carbon sinks was established based on the accounting method of ecosystem GDP. Third, the economic value of the shellfish and algae carbon sinks was comprehensively calculated using indicators including product value, carbon storage value, and oxygen release value. Fourth, the carbon sink potentials of shellfish and algae cultures were evaluated. Finally, ways to expand the carbon sink capacities of shellfish and algae cultures were proposed with the aim of providing a reference that will help countries fulfill their commitment to carbon neutrality, actively participate in global carbon emission control, and achieve sustainable development.

## 2. Materials and Methods

### 2.1. Data Sources

Data on shellfish and algae mariculture output in China from 2003 to 2019 were taken from the statistical yearbook of China’s fisheries issued by the Ministry of Agriculture and Rural Areas of the People’s Republic of China.

**Figure 1:**
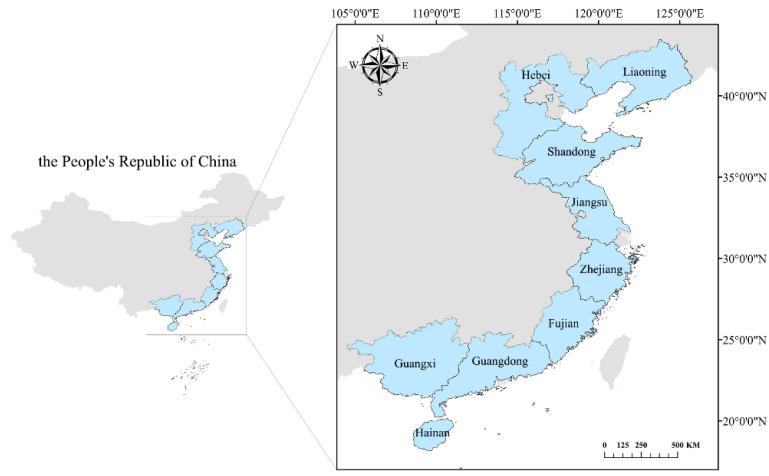
Map of China’s coastal areas. The shellfish and algae mariculture production in Hong Kong, Macao, Taiwan Province, Shanghai, and Tianjin were not included due to data availability and validity concerns.

According to the availability of data, this study excluded Hong Kong, Macao, and the Taiwan Province of China. In the statistical yearbook of China’s fisheries, the outputs of shellfish and algae mariculture in Shanghai and Tianjin were 0; therefore, based on the validity of the data, this study also excluded Shanghai and Tianjin. In total, this study retained the data from 9 provinces, including Jiangsu Province, Shandong Province, etc. (Figure 1). Mariculture in China mainly includes fish, shellfish, crustaceans, algae, and others. The mariculture carbon sink in this study was simplified to mainly include shellfish and algae because the bait is used in the cultures of fish and crustaceans, while carbon sink fisheries are non-bait fisheries. The species of shellfish used in mariculture include *Crassostrea gigas*, *Abalone*, *Busycon canaliculatu*, *Scapharca subcrenata*, *Mytilus edulis*, *Pinna rudis* Linnaeus, *Chlamys farreri*, *Ruditapes philippinarum*, and *Sinonovacula constricta*, while those of algae include *Laminaria japonica*, *Undaria pinnatifida Suringar*, *Porphyra*, *Gracilaria ferox*, *Eucheuma muricatum*, *Gelidium amansii* Lamouroux, *Hizikia fusiforme*, and *Enteromorpha prolifra*.

The economic value of the shellfish and algae carbon sink from 2003 to 2019 was calculated using the exchange rate of USD to RMB in 2019 (1:6.88). According to the statistics of the National Fisheries Technology Extension Center and the China Society of Fisheries, the average market price of mariculture products in China in 2019 was RMB 43.3/kg, or USD 6.30/kg. Due to the lack of market prices of shellfish and algae from 2003 to 2018, the “production price index of mariculture products” in the *Yearbook of China’s Agricultural Product Price Survey* published by the National Bureau of Statistics of the People’s Republic of China was used as the price index for derivation and calculation, taking 2003 as the base period.

### 2.2. Assessment of Carbon Sink Capacity of Shellfish and Algae

The carbon sink capacity of shellfish and algae was evaluated using the material quality assessment method based on the “yield–carbon sink coefficient–carbon sink” relationship of marine organisms. The assessment method for the carbon sink capacity of shellfish and algae is as follows:

(1) $$C_{mariculture}=C_{shellfish}+C_{macroalgae}\;$$

where $C_{mariculture\;}$ represents the total capacity of the carbon sink of shellfish and algae (t/year), $C_{shellfish}$ represents the carbon sink capacity of shellfish (t/year), and $C_{macroalgae}$ represents the carbon sink capacity of algae (t/year).

The assessment method of the carbon sink capacity of shellfish is as follows:

(2) $$C_{shellfish}=\overset{n}{\underset{i}{\sum }}\left(CB_{i}^{sh}+CZ_{i}^{sh}\right)$$

where $CB_{i}^{sh}$ represents carbon sink capacity of the shells of different shellfish (t/year), and $CZ_{i}^{sh}$ represents carbon sink capacity of the soft tissues of different shellfish (t/year).

The assessment method of the carbon sink capacity of shellfish shells is as follows:

(3) $$CB_{i}^{sh}=P_{i}^{sh}\times K_{i}^{sh}\times R_{i}^{sh1}\times CF_{i}^{sh1}$$

where $P_{i}^{sh}$ represents the wet weight biomass of different shellfish (t/year), $K_{i}^{sh}$ represents the conversion coefficient between wet weight and dry weight of different shellfish, $R_{i}^{sh1}$ represents the proportion of the dry weight made up of shell dry mass for different shellfish, and $CF_{i}^{sh1}$ represents the carbon content to dry mass ratio of the shells of different shellfish.

The assessment method of the carbon sink capacity of shellfish soft tissues is as follows:

(4) $$CZ_{i}^{sh}=P_{i}^{sh}\times K_{i}^{sh}\times R_{i}^{sh2}\times CF_{i}^{sh2}$$

where $R_{i}^{sh2}$. represents the proportion of dry weight made up of soft tissue dry mass for different shellfish, and $CF_{i}^{sh2}$ represents the carbon content ratio of soft tissue to dry mass of different shellfish. The assessment parameters of shellfish carbon sink capacity [13,26,27] are shown in Table 1. *Abalone*, which is not a filter-feeding shellfish, was not included in the calculation of the carbon sink of shellfish culture.

The assessment method of the carbon sink capacity of algae is as follows:

(5) $$C_{macroalgae}=\overset{n}{\underset{j}{\sum }}\left(P_{j}^{ma}\times K_{j}^{ma}\times CF_{j}^{ma}\right)\;$$

where $P_{j}^{ma}$ represents the wet weight biomass of different algae (t/year), $K_{j}^{ma\;}$ represents the conversion coefficients between wet weight and dry weight for different algae, and $CF_{j}^{ma}$ represents the carbon content ratio of the dry mass of shells of different algae. The assessment parameters of algae carbon sink capacity [13,28,29,30] are shown in Table 2.

### 2.3. Accounting for the Economic Value of the Shellfish and Algae Carbon Sinks

The mariculture of shellfish and algae serves multiple functions, acting as supplies of oxygen and commercial products, as a carbon sink, and as a regulator of the carbon system equilibrium. The economic value of the shellfish and algae aquaculture can be divided into the value of the product, the carbon storage value, and the value of the oxygen released by algae during growth, which can be estimated using the ecosystem GDP accounting method. The accounting method for determining the economic value of the shellfish and algae carbon sink is as follows:

(6) $$V_{mariculture}=V_{p}+V_{c}+V_{o}$$

where $V_{mariculture}$ represents the overall economic value of the shellfish and algae carbon sink of (USD/year), $V_{p}$ represents the product value (USD/year), $V_{c}$ represents the carbon storage value (USD/year), and $V_{o\;}$ represents oxygen release value (USD/year).

The market value method adopted to determine product values is as follows:

(7) $$V_{p}=\overset{n}{\underset{i}{\sum }}\left(Q_{i}\times P_{i}\right)$$

where $Q_{i}$ represents the yield of a specific shellfish or algae (t/year), and $P_{i}$ represents the market price of that shellfish or algae (USD/t). This study adopted the market prices of mariculture products.

The market value method for assessing carbon storage values is as follows:

(8) $$V_{c}=C_{mariculture}\times k_{1}\times P_{C}$$

where $k_{1}$ represents the mass conversion coefficient between carbon and CO_2_ (44/12), and $P_{C}$ represents the carbon tax rate proposed by the Swedish government (USD 150/t).

The alternative cost method adopted for the value of oxygen release is as follows:

(9) $$V_{o}=C_{macroalgae}\times k_{2}\times C_{I}$$

where $C_{macroalgae}$ represents the carbon sink capacity of algae (t/year), $k_{2}$ represents the mass conversion coefficient of carbon and oxygen (32/12), and $C_{I}$ represents the cost of industrial oxygen production (USD/t), which is calculated according to the oxygen production cost of PSA oxygen production equipment (USD 122/t) [31].

## 3. Results

### 3.1. Status of Shellfish and Algae Culture

**Figure 2:**
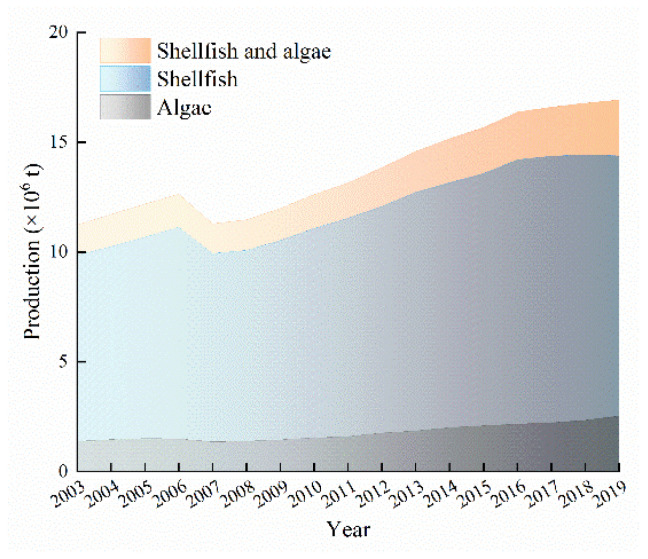
Cumulative output of shellfish and algae mariculture in China from 2003 to 2019.

The cumulative output of shellfish and algae mariculture in China from 2003 to 2019 exceeded 230 million tons and had an average annual output of 13.78 million tons (Figure 2), increasing by 50.65% from 2003 (11.24 million tons) to 2019 (16.93 million tons). With the exception of 2007, the output increased by varying degrees year after year from 2003 to 2019 with annual growth rates ranging from 0.86% to 5.35% and an average annual growth rate of 3.55%. The highest annual growth rates occurred from 2005 to 2006 and from 2009 to 2014, both of which exceeded 4.00%. Multiple extreme weather events occurred in the coastal areas of China in 2007, including a warm winter and abnormally high temperatures, drought, snow disaster, and extreme temperate storm surge. Specifically, the most serious temperate storm surge disaster to occur in China since 1969 occurred in the Bohai and the Yellow Sea on 3 March 2007, resulting in a 10.64% decrease in the total output of shellfish and algae mariculture, compared with the previous year.

**Figure 3:**
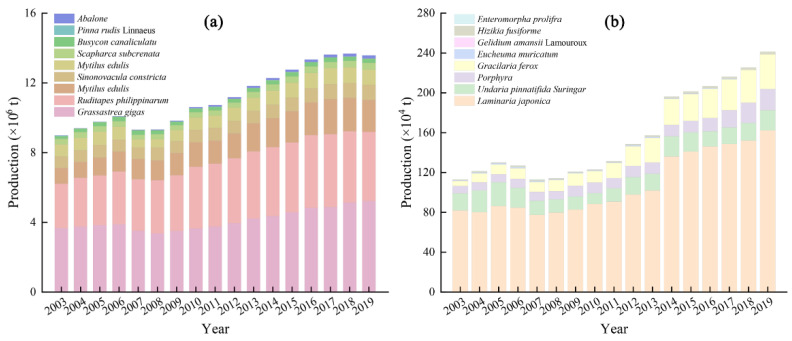
Output of (**a**) shellfish mariculture and (**b**) algae mariculture in China from 2003 to 2019.

Shellfish mariculture made up the majority of the total output of shellfish and algae mariculture, with an annual average proportion of 87.35%. From 2003 to 2019, the cumulative output of shellfish mariculture in China exceeded 200 million tons, with an average annual output of 12.03 million tons. *Crassostrea gigas*, *Ruditapes philippinarum*, and *Chlamys farreri* were the main species of marine shellfish culture in China (Figure 3a) and together amounted to 80.81% of the total shellfish output.

The average annual output of algae mariculture in China from 2003 to 2019 was 1.78 million tons, accounting for 12.65% of the total output of shellfish and algae mariculture. *Laminaria japonica*, *Undaria pinnatifida Suringar*, *Porphyra*, and *Gracilaria ferox* were the main algae species cultured in China (Figure 3b) and together accounted for 98.55% of the total output of algae, with *Laminaria japonica* being most popular and accounting for 68.44% of the algae output.

### 3.2. Carbon Sink Capacity of Shellfish and Algae Mariculture

**Figure 4:**
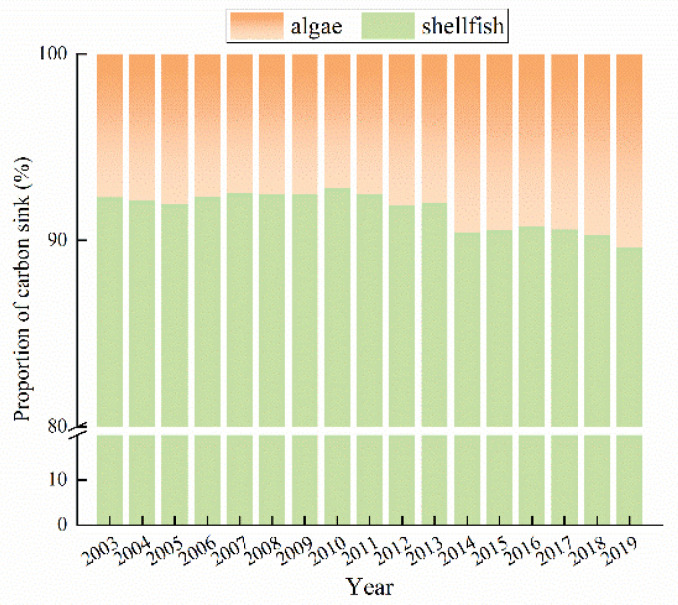
Proportion contributions to the fisheries carbon sink by shellfish and algae in China from 2003 to 2019.

In total, shellfish and algae mariculture in China from 2003 to 2019 represented a carbon sink that accumulated more than 18 million tons, with an average annual carbon sink of 1.10 million tons (Figure 4). Furthermore, the annually sequestered carbon increased by 54.1% over the study period, from 893,400 tons in 2003 to 1,360,500 tons in 2019. Generally, the carbon sink increased in size each year from 2003 to 2019, except for a period of fluctuation from 2006 to 2010.

Shellfish dominated the total carbon sink comprising shellfish and algae mariculture, accounting for 91.63% of the annual average carbon sequestered; however, this proportion decreased from 2014 to 2019 and dropped to 89.61% in 2019. Conversely, the proportion of the carbon sink contributed by algae mariculture increased from 2014 to 2019. Consistent with the proportion of output, *Crassostrea gigas*, *Ruditapes philippinarum*, and *Chlamys farreri* were the main contributors to the shellfish carbon sink. They represented average annual carbon sinks of 394,900 tons, 224,200 tons, and 147,100 tons, respectively, and their average annual contributions were 39.36%, 22.32%, and 14.44%. *Laminaria japonica* was the main algal contributor to the carbon sink, responsible for 72.05% on average of all algal sequestration, and sequestering 67,500 tons of carbon on average each year.

### 3.3. Economic Value of the Shellfish and Algae Carbon Sinks

The cumulative economic value of the shellfish and algae carbon sink in China from 2003 to 2019 exceeded USD 1,210,000 million, and the annual average economic value of the carbon sink was USD 71,303.56 million (Table 3). The economic value of the shellfish and algae carbon sink increased by 201.29%, from USD 35,143.61 million in 2003 to USD 106,637.33 million in 2019, and showed a stable growth trend year after year, except for in 2007. Product value was the main contributor to the total economic value of the carbon sink with an annual average proportion of 99.11%, while the average annual proportions of the carbon storage and oxygen release values were 0.85% and 0.04%, respectively.

## 4. Discussion

### 4.1. Carbon Sink Conversion Efficiency of Shellfish and Algae

The shellfish and algae mariculture in China output an average of 13.78 million tons from 2003 to 2019 and represented an average annual carbon sink of 1.10 million tons. These values indicated that there was a problem in the carbon sink conversion efficiency. The conversion ratio of a carbon sink is not only an important basis on which to measure carbon sink capacity but also a comprehensive embodiment of carbon sink technology and the sustainability of the mariculture industry [19,32,33]. In mariculture, when the practices and technologies for carbon separation, fixation, and recovery are developed and enriched, less carbon is lost, and the carbon sink conversion ratio and carbon sink capacity are increased. For example, at the marine biological farm in Shandong Province, China, to maximize its carbon sink role, save breeding space, and improve the quality and output of aquatic products, they use optimized three-dimensional breeding spaces and layered breeding practices. Specifically, the surface layer is used for the in situ restoration of an ecological floating bed, the middle layer is used for raising fish and shrimp, and shellfish and algae are cultured in the bottom layer [34].

The carbon sink conversion ratio of shellfish and algae mariculture in this study was defined as the ratio of the shellfish and algae carbon sink to their total output. The carbon sink calculation in this study can be seen as a removable carbon sink based on aquaculture output and the carbon content in aquaculture organisms, without considering the feed cost and energy cost of shellfish and algae mariculture. The CO_2_ emissions per unit of protein produced by marine shellfish and algae culture generally compare favorably with most livestock production and some wild-caught fisheries. The lower emission intensity is mostly attributable to a more favorable feed conversion ratio [11]. The primary mariculture organisms in China include macroalgae and filter-feeding shellfish, which do not feed or feed at a low trophic level, making their culture structures relatively stable. In terms of energy costs, “blue” biofuels, such as seaweed biofuels, do not compete for resources with agriculture, as they do not require arable land, freshwater or fertilizer, or herbicide or pesticide applications and are, therefore, in many respects, more environmentally sustainable than current biofuels derived from land crops, which can be considered as another form of emission and energy reduction [16]. Meanwhile, the calculation of removable carbon sink does not consider the process by which shellfish and algae are consumed by consumers. In the case of algae, if used for food, they are quickly reconverted to CO_2_ and energy, preventing long-term carbon sinks [14]. However, algae-based food systems can replace food or feed production systems with intense CO_2_ emission footprints because they have much lower life-cycle CO_2_ emissions. For shellfish, the shell is usually discarded when tissue is consumed and respired, and these shells act as long-term carbon stores.

The carbon sink conversion ratios of shellfish and algae from 2003 to 2019 in China were 8.37% and 5.20%, respectively, which is mainly caused by the fundamental differences in their biological structures [35,36]. As aquatic plants, the conversion coefficient of algae between wet weight and dry weight is 20% because they have higher water content than shellfish; furthermore, their carbon contents are relatively low. For mollusks, soft tissues generally account for less than 10% of the total weight, and they have a high dry weight ratio, large total weight ratio, and high carbon content, leading to a higher carbon sink conversion efficiency than algae. Due to the massive scale of marine aquaculture and the high carbon sink conversion ratio of shellfish, the shellfish aquaculture industry has grown to have the largest carbon sink capacity and greatest potential in China. Notably, the most important factor affecting the carbon sink conversion ratio in aquaculture is how the culture is structured with different marine organisms. Although the algae culture structure has a lower carbon sink conversion ratio, algae play roles in improving water quality, providing oxygen, and facilitating the growth of aquatic animals, and their ecological service and positive external effect on the environment cannot be ignored [37,38]. Therefore, increasing the scale of shellfish and algae mariculture will guarantee the improvement of the mariculture carbon sink [13]. On this basis, optimizing the mariculture structure can further maximize the carbon sink capacity of mariculture.

### 4.2. Carbon Sink Potential of Shellfish and Algae

**Figure 5:**
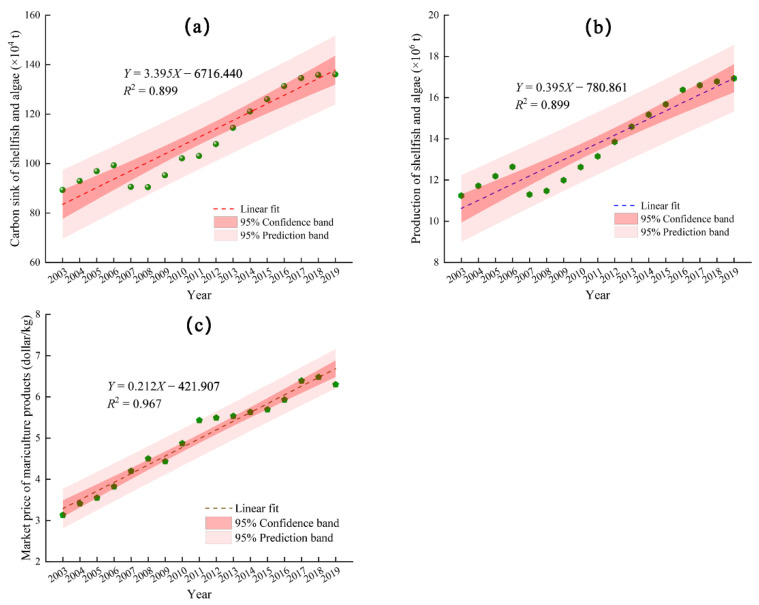
(**a**) Linear trend of the total shellfish and algae carbon sink over time; (**b**) linear trend of the total shellfish and algae mariculture production over time; (**c**) linear trend of the production price index of mariculture products over time.

The linear trend in the carbon sink capacity of shellfish and algae mariculture year after year was analyzed to evaluate the future carbon sink potential of shellfish and algae mariculture in China (Figure 5a). The slope of the trend line indicated that the shellfish and algae carbon sink grew by 33,900 tons/year, with the growth rates of shellfish and algae being 28,100 tons/year and 4700 tons/year, respectively. In 2020, China promised to adopt more effective policies and measures that strive for peak CO_2_ emissions by 2030 and to achieve carbon neutrality by 2060. It is assumed that the growth rate of shellfish and algae mariculture production in China will be stable over the next few decades, and as such, the shellfish and algae carbon sink in China will grow to 1,751,700 tons/year in 2030, representing a 27.02% increase over 2019. Over that same period, the forest area of China will reach 196.21 × 10^6^ hectares, with a carbon sink capacity of 230.15 million tons/year [39], so it can be calculated that the carbon sink from harvesting shellfish and algae in 2030 will be equivalent to the carbon fixed by 0.15 × 10^6^ hectares of forest. The carbon sink capacity of wetlands in China is 315.76 tons per hectare [40], which means that the carbon sink achieved by harvesting shellfish and algae in 2030 will be equivalent to the carbon fixed by 5546.93 hectares of wetlands. In 2060, the shellfish and algae carbon sink in China will be 2.77 million tons/year, an increase of 100.88% compared with 2019.

The total output of shellfish and algae mariculture (Figure 5b) and the market price of mariculture products (Figure 5c) were also predicted assuming steady growth. The linear slope indicated that the total output of shellfish and algae mariculture in China increased by 395,100 tons/year. In 2030, the total output of shellfish and algae in China will be 21.29 million tons/year, a 25.75% increase over 2019. In 2060, the total output of shellfish and algae in China will be 33.14 million tons/year, a 95.78% increase over 2019. The market prices of mariculture products in 2030 and 2060 were predicted to be USD 9.02 and USD 15.39, respectively. These prices assumed that the carbon tax rate, cost of industrial oxygen production, and the exchange rate of USD against RMB will remain unchanged. The value of the shellfish and algae carbon sink in 2030 and 2060 will be USD 191,978.44 million and USD 510,046.47 million, respectively. Moreover, the carbon storage value in 2030 and 2060 will be USD 963.42 million and USD 1523.57 million, respectively, and the oxygen release value will be USD 59.49 million and USD 105.35 million, respectively. The economic value of the shellfish and algae carbon sink in 2030 and 2060 will be USD 193,001.35 million and USD 511,675.39 million, respectively. These trends indicate that the potential of the shellfish and algae carbon sink in China is huge and can bring considerable economic benefits and make an important contribution to the grand goal of carbon neutralization in China.

The carbon sink calculation in this study was based on the biomass of harvested shellfish and algae mariculture products, which can be seen as a removable carbon sink based on aquaculture output and the carbon content in aquaculture organisms [41]. The removable carbon sink of algae mainly comes in the form of biomass carbon fixed via photosynthesis, while that of shellfish mainly refers to the calcium carbonate shells and soft tissues formed during shellfish growth. In fact, the carbon fixed by shellfish and algae mariculture includes three main forms: the removable carbon sink, the carbon that remains in seawater in the forms of particulate organic carbon (POC) and dissolved organic carbon (DOC), and carbon buried in sediments [42].

Detrital organic carbon produced during algal growth can be a food source for other organisms through the natural food chain, deposited and buried on the seafloor, or transported to the deep sea through direct sedimentation [43,44,45]. DOC and POC released by macroalgae during growth can enter the food web or form recalcitrant DOC (RDOC) through microbial cycling, which will remain in seawater for a long time [46,47]. Marine shellfish have a great impact on the biogeochemical cycle of carbon because they interact with phytoplankton, particulate organic debris, seawater carbonate system, sedimentation, and burial through the processes of filtration, respiration, calcification, and biological deposition [48]. Cultured shellfish use carbon in the ocean in two main ways: directly converting the ${\mathrm{HCO}}_{3}^{-}$ in seawater into CaCO_3_ for shells through calcification and through the synthesis of their own body mass by filtering POC (phytoplankton, microzooplankton, organic debris, microorganisms, etc.) from the water body. Unused organic carbon settles on the seafloor in the form of fecal particles, which accelerates the transportation of organic carbon to the seafloor [49]. Therefore, carbon can be removed from seawater both by harvesting cultured shellfish and through the BP and carbonate pump driven by cultured shellfish. The mechanisms underlying the shellfish and algae mariculture carbon sink are extremely complex [6,7,8,9,10] and have recently been proposed to involve the traditional solubility pump, carbonate pump, BP, and MCP. The fishery carbon sink represented by cultured shellfish and algae has been studied from the initial “removable carbon sink” to the deposition and burial of POC and the formation of RDOC in water. The study of the organic carbon pool in coastal aquaculture areas is particularly important for human beings when exploring the “missing carbon sink” in shallow water [50]. However, scientific measurement methods for the shellfish and algae carbon sink have not yet been fully formed [51,52], so in the present study, we only considered “removable carbon sink”, which, in fact, underestimates the shellfish and algae carbon sink.

### 4.3. Amplification of Carbon Sink Capacity of Shellfish and Algae

Although carbon sequestration by shellfish and algae culture appears to be growing, it is undeniable that the culture structure and scale of shellfish and algae are subject to various fluctuating factors such as climate, niche, space, and demand, which results in uncertainty in predicting carbon sequestration in the future [16,23]. In particular, conflicts between the industry’s need for space and marine wetland ecological restoration [53] and conservation in coastal provinces, as well as competition with other forms of mariculture, will limit the potential for shellfish and algae culture structure and its development, thereby affecting the potential growth in the carbon sink capacity in the future. However, this study shows that expanding carbon sequestration via shellfish and algae culture should be prioritized because it is an environmentally important industry that produces in-demand products and is economically profitable [48].

The carbon sequestration pathways in an aquaculture environment mainly include the burial of POC from BP processes in the sediment of the aquaculture area, RDOC formed by MCP processes, and carbon deposition into the deep sea [42,46,47]. There are various ways to improve carbon sequestration. For example, the activities of BP and MCP can be enhanced via artificial upwelling and comprehensive culture of shellfish and algae, which will subsequently improve carbon sequestration in offshore and estuarine culture areas. Within the scope of the mariculture industry, there are numerous approaches that can improve the shellfish and algae carbon sink.

(1) Expand breeding space and improve breeding yield

The amount of removable carbon accumulated in shellfish and algae culture is positively correlated with the yield of cultured shellfish and algae per unit area and the carbon content per organism. Therefore, increasing the yield per unit area and screening for shellfish and algae with higher individual carbon contents are potential ways to improve carbon sequestration. By breeding varieties with high carbon sequestration rates, improving breeding technology and mode, and making rational and efficient use of sea breeding areas, the yield per unit area can be increased, which will increase the amount of removable carbon per unit area. Furthermore, breeding strains that can break out of their conventional growth environments possibly strains with wider temperature tolerance ranges, would broaden the potential growing regions for specific species. This would be another effective way to increase the potential carbon sequestration capacity of shellfish and algae mariculture [54].

(2) Promote multi-trophic-level, comprehensive breeding models

In a reasonable proportion of shellfish and algae polyculture systems, algae not only absorb nitrogen, phosphorus, and other nutrients released by shellfish metabolism but also absorb CO_2_ released by shellfish respiration. Shellfish feed by filtering phytoplankton, algal debris, and litter from the seawater. This benefits commercial algae species in two ways: It purifies the water, thus increasing water illumination and providing more energy for algal growth, and it prevents phytoplankton from competing with algae for nutrients, which is conducive to the growth and carbon accumulation of cultured algae [55,56]. Through this shellfish–algae interaction, the carbon sink function of the whole integrated culture system is greatly improved, compared with single species cultures [57,58].

(3) Implementation of artificial marine upwelling exchange enhancement project

The application of artificial upwelling technology to transport excess nutrients from deep water to surface waters can fully meet the requirements for photosynthetic carbon sequestration and the growth of algae [59]. Appropriate nutrient concentrations not only increase the output of seaweed but also improve the comprehensive effect of the biological and microbial carbon pumps, so as to increase the offshore carbon sink. As a geoengineering system, artificial upwelling can continuously transport the deep-water layer from below the true light layer into the euphotic zone where algal growth can occur. When saline seawater is brought to the true light layer, it not only increases the total nutrient concentrations but also compensates for imbalances in the proportions of nitrogen, phosphorus, silicon, and iron caused by biological growth, utilization, and release. These nutrients are conducive to photosynthesis by algae and phytoplankton, which increases production and the aquaculture carbon sink, as well as the organic carbon output to the deep sea by increasing the efficiency of the biological pump [60,61].

In addition to technology, policies should be implemented to guide market players to expand the scale of shellfish and algae breeding, support innovative breeding modes, improve the degree of intensive breeding, actively explore the diversification of shellfish and algae processing, improve storage and consumption modes, and cultivate and expand the consumer market for shellfish and algae products. At the same time, we should carry out research on the pricing mechanisms and standard formulations of fishery carbon sequestration and explore the establishment of a fishery carbon sequestration listing and trading system to promote the paid ecological service of aquaculture fishery carbon sequestration and realize the full carbon sequestration potential of these marine fisheries.

## 5. Conclusions

Based on the “yield–carbon sink coefficient–carbon sink” relationship of marine organisms, in this study, we evaluated the capacity and potential of the shellfish and algae carbon sink in China. The economic value of shellfish and algae carbon sink was comprehensively calculated using important indicators, i.e., product value, carbon storage value, and oxygen release value. Shellfish contributed 91.63% to the total shellfish and algae carbon sink. The main shellfish species were *Crassostrea gigas*, *Ruditapes philippinarum*, and *Chlamys farreri*, contributing averages of 39.36%, 22.32%, and 14.44% to the annual carbon sequestration capacity, respectively. The annual average economic value of the shellfish and algae carbon sink in China was USD 71,303.56 million, and the product value constituted most of the total, accounting for 99.11% on average. The carbon sink conversion ratios of shellfish and algae in China were 8.37% and 5.20%, respectively, which meant that the shellfish aquaculture industry had the strongest carbon sink capacity in China, along with the greatest growth potential. The growth rate of the removable carbon sink of shellfish and algae culture in China was 33,900 tons/year during the study period, but this trend is not guaranteed. In the future, the capacity for carbon sequestration and exchange by aquaculture practices can be improved by expanding breeding spaces, promoting multi-level, comprehensive breeding modes, and marine artificial upwelling projects. This study provides an important reference that will help countries realize carbon neutrality commitment, as well as actively drive movement toward global carbon emission governance and sustainable development.

## Figures and Tables

**Table 1:** Assessment parameters of shellfish carbon sink capacity.

Species	$K_{i}^{sh}\;(\%)$	Dry Mass Specific Gravity		Carbon Contents (%)	
		$R_{i}^{sh1}\;(\%)$	$R_{i}^{sh2}\;(\%)$	$CF_{i}^{sh1}\;(\%)$	$CF_{i}^{sh2}\;(\%)$
*Crassostrea gigas*	65.10	93.86	6.14	12.68	45.89
*Busycon canaliculatu*	64.21	87.91	12.09	11.98	44.99
*Scapharca subcrenata*	64.21	53.47	46.53	11.29	45.86
*Mytilus edulis*	75.28	91.53	8.47	11.76	44.40
*Pinna rudis* Linnaeus	64.21	88.59	11.41	11.44	43.87
*Chlamys farreri*	63.89	85.65	14.35	11.40	43.90
*Ruditapes philippinarum*	52.55	98.02	1.98	11.52	44.90
*Sinonovacula constricta*	70.48	96.74	3.26	13.24	44.99

**Table 2:** Assessment parameters of algae carbon sink capacity.

Species	$K_{j}^{ma}\;(\%)$	$CF_{j}^{ma}\;(\%)$
*Laminaria japonica*	20.00	31.20
*Undaria pinnatifida Suringar*	20.00	30.70
*Porphyra*	20.00	27.39
*Gracilaria ferox*	20.00	20.60
Others ^1^	20.00	27.76

^1^ Other algae include *Eucheuma muricatum*, *Gelidium amansii Lamouroux*, *Hizikia fusiforme*, and *Enteromorpha prolifra*.

**Table 3:** Economic value of the shellfish and algae carbon sink from 2003 to 2019.

Year	Market Price of Mariculture Products	Economic Value/Million Dollars	Product Value		Carbon Storage Value		Oxygen Release Value	
			Value Quantity/Million Dollars	Proportion /%	Value Quantity/Million Dollars	Proportion /%	Value Quantity/Million Dollars	Proportion/%
2003	3.13	35,657.37	35,143.61	98.56	491.39	1.38	22.37	0.06
2004	3.41	40,469.99	39,934.99	98.68	511.25	1.26	23.74	0.06
2005	3.55	43,805.81	43,247.36	98.73	532.98	1.22	25.47	0.06
2006	3.82	48,839.94	48,269.19	98.83	546.02	1.12	24.73	0.05
2007	4.20	47,992.30	47,472.30	98.92	498.05	1.04	21.95	0.05
2008	4.50	52,073.67	51,554.21	99.00	497.31	0.96	22.15	0.04
2009	4.43	53,593.21	53,046.02	98.98	523.83	0.98	23.36	0.04
2010	4.87	62,096.86	61,511.48	99.06	561.50	0.90	23.88	0.04
2011	5.43	71,993.01	71,400.78	99.18	566.89	0.79	25.34	0.04
2012	5.49	76,604.59	75,982.69	99.19	593.45	0.77	28.45	0.04
2013	5.53	81,263.03	80,603.59	99.19	629.56	0.77	29.88	0.04
2014	5.63	86,168.12	85,464.43	99.18	666.01	0.77	37.68	0.04
2015	5.69	89,912.11	89,180.51	99.19	692.93	0.77	38.67	0.04
2016	5.93	97,814.06	97,052.34	99.22	722.18	0.74	39.55	0.04
2017	6.39	106,873.66	106,092.27	99.27	740.12	0.69	41.27	0.04
2018	6.48	109,571.06	108,780.98	99.28	747.13	0.68	42.95	0.04
2019	6.30	107,431.64	106,637.33	99.26	748.28	0.70	46.03	0.04
Total	/	1,212,160.44	1,201,374.08	/	10,268.89	/	517.47	/

The average market price of mariculture products in China in 2019 was USD 6.30/kg. The “production price index of mariculture products” in the *Yearbook of China’s agricultural product price survey* published by the National Bureau of Statistics of the People’s Republic of China was used as the price index for deriving and calculating prices for other years, taking 2003 as the base period.

## Data Availability

The data and software generated or used during the study appear in the submitted article.
